# Systemic juvenile idiopathic arthritis: frequency and long-term outcome in Western Australia

**DOI:** 10.1007/s00296-023-05318-1

**Published:** 2023-03-29

**Authors:** Johannes C. Nossent, Erin Kelty, Helen Keen, David Preen, Charles Inderjeeth

**Affiliations:** 1 Department of Rheumatology, Sir Charles Gairdner Hospital, Perth, Australia; 2 Rheumatology Group, School of Medicine, University of Western Australia, 35 Stirling Highway (M503), Perth, Australia; 3 School of Population and Global Health, University of Western Australia, Nedlands, Perth, Australia; 4 Fiona Stanley Hospital, Perth, Australia

**Keywords:** Systemic juvenile arthritis, Epidemiology, Linked health data, Comorbidity, Outcome

## Abstract

Systemic juvenile idiopathic arthritis (S-JIA) is a rare but potentially life threatening autoinflammatory condition of childhood. Given the limited data on S-JIA from the Australasian region, we investigated the epidemiological characteristics and long-term disease outcome in S-JIA. All hospitalised patients under the age of 16 years registered with ICD-10-AM code M08.2 in in the period 1999–2014 were identified in longitudinally linked administrative health data across all Western Australian (WA) hospitals. Incidence and point prevalence estimate were per 100,000 population with Poisson regression to analyse the incidence trend. Readmissions with S-JIA as primary diagnosis were considered flares with rates for flare and other complication reported per 100 person years with 95% confidence intervals (CI). Annual S-JIA incidence was 0.61/100,000 (CI 0.28–1.25) (46 incident cases, 71.7% girls, median age 6.5 years) and stable over time as S-JIA point prevalence reached 7.15/100,000 (CI 5.29–7.45) at the end of study. Most incident cases were diagnosed in winter and spring, but documented preceding infections were rare. During a median follow-up of 8 years, disease flares occurred in 24% of patients with higher flares rate in boys (58.3; CI 44.5–74.9) than girls (14.7; CI 9.9–20.9). No deaths occurred and arthroplasty was the main, but uncommon S-JIA complication (4%). However, readmission (86.3; CI 76.4–97.2) and ED visit (73.3; CI 64.2–83.4) rates for illnesses other than S-JIA were substantial. S-JIA is as rare in WA as in other regions and while s-JIA incurred no deaths in the era of biologics, it associated with a significant long-term burden of (co-) morbidity.

**Supplementary Information:**

The online version contains supplementary material available at 10.1007/s00296-023-05318-1.

## Introduction

Systemic juvenile idiopathic arthritis (S-JIA) also known as Still’s disease (SD) is a rare systemic inflammatory disease of unknown origin appearing before 16 years of age. S-JIA typically presents with a combination of daily fever spikes, evanescent rash and arthritis in association with leucocytosis and elevated acute phase reactants, while organ manifestations can include lymphadenopathy, hepatosplenomegaly, serositis and myocarditis [1, 2]. As there is no diagnostic test or marker, S-JIA remains a diagnosis of exclusion and is usually first considered when treatment of suspected infections fails. Both S-JIA and its counterpart in adult patients (AOSD) are uncommon conditions [3] considered to be autoinflammatory diseases, where an unresolved combination of genetic predisposition and exogenous triggers leads to uncontrolled production of proinflammatory cytokines [2, 4, 5]. Although corticosteroids are still considered the first line treatment for S-JIA, their short and long-term side effects have led to increasing use of anti-cytokine drugs for S-JIA [6, 7]. Despite this, the disease course remains unpredictable as S-JIA appears self-limiting after initial treatment in some patients but leads to recurrent exacerbations of systemic inflammation and/or development of chronic deforming arthritis in others [8, 9]. With scarce data available on S-JIA from the Australasian region, we investigated the epidemiology and longitudinal outcomes in hospitalised S-JIA patients in Western Australia (WA) in the period 1999–2014.

## Methods

This was population-level observational study includes children under the age of 16 with a recorded first diagnosis of S-JIA (ICD-10-AM M08.20-M08.29) residing in WA between January 1999 and December 2014. Data were derived from the WA Rheumatic Disease Epidemiological Registry (WARDER) that contains routinely collected health data from public and private health care organisations for the entire state of WA for patients with rheumatic diseases. Sourced from the Hospital Morbidity Data Collection (HMDC), Emergency Department Data Collection (EDDC) (data from 2002 onwards), the WA Cancer Register and the WA Death Register, these datasets are effectively linked through a validated process of probabilistic matching and clerical review to provide individual longitudinal health data. The final dataset contained sociodemographic data, all principal and secondary diagnoses for all hospital contacts for each participant in addition to information on principal and secondary procedures performed, length and type of admission (e.g., intensive care) and diagnostic codes for any ED visit, ever recorded cancer type and time and cause of death during the observation period.

### Outcome ascertainment

We defined time-zero (T0) as the date of S-JIA diagnosis and the follow up period as all observation time > 30 days after T0. Readmission with S-JIA as the primary diagnosis was considered a disease flare, while serious infections were defined as episodes leading to ED presentation and/or hospital admission resulting in an infectious disease code [3]. Death was ascertained through the WA Death Register and arthrocentesis, arthroplasty, diabetes mellitus, osteoporosis and fractures through the relevant diagnostic and procedure codes (suppl Table 1).

### Statistical analyses

Descriptive statistics are presented as median plus interquartile range (IQR) for numeric variables and proportions for categorical variables, unless otherwise indicated. Differences for numeric results were compared by non-parametric methods (Kruskal–Wallis) and for proportions by Chi-square with Yates correction where needed. Average annual incidence and point prevalence rates are given per 100,000 population with the total number of cases as numerator and a denominator for the population < 16 years in that year. Historical population data for WA were obtained from the Australian Bureau of Statistics (https://www.abs.gov.au/statistics/people/population/national-state-and-territory-population/latest-release#data-downloads-data-cubes). A generalized log-linear regression model (Poisson) was used to analyse the trend in the number of cases per year. All-cause hospitalisation and ED visit rates (expressed as number per 100 person-years at risk) with 95% Confidence Intervals (CI). Analyses were performed using SPSS v27.0 (IBM, USA) and Open-Epi software with two-sided *p* values < 0.05 considered to be statistically significant.

### Ethics

This project was approved by the Human Research Ethics Committee at the WA Department of Health (HREC nr 2016.24) with the condition to prevent potential identification by confidentializing small numbers (*n* < 5).

## Results

**Fig. 1:**
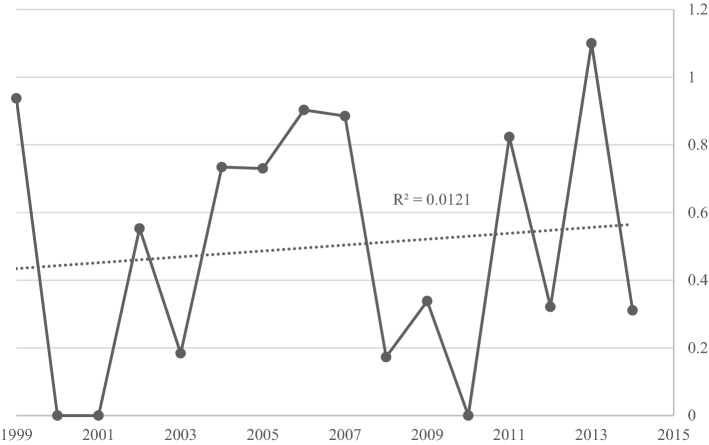
Annual incidence rate per 100.000 for Systemic juvenile idiopathic arthritis (S-JIA) in Western Australia over study period

A total of 46 patients were hospitalised with incident S-JIA in the study period, including 33 girls (71.7%) and 13 boys (28.3%). The average annual incidence rate for S-JIA was 0.61/100,000 (CI 0.28–1.25), which did not no change significantly over the 15-year period (Fig. 1) leading to a point prevalence of S-JIA in 2014 of 7.15/100,000 (CI 5.29–7.45).

Most incident cases were diagnosed in winter and spring (Suppl Fig. 1), although there was a low rate of documented preceding infections (Table 1).

**Table 1:** Patient characteristics at incident hospitalisation for systemic juvenile idiopathic arthritis

	All (*n* = 46)	Girls (*n* = 33)	Boys (*n* = 13)	*p* value
Age at diagnosis	6.5 (3.8–11) (3.8–1111)	7 (5–12)	6 (2–10)	0.17
Previous injury/poisoning	11 (23.9)	9 (27.7)	< 5 (15.4)	0.81
Preceding infections				
Pneumonia	< 5 (8.7)	< 5 (3.0)	< 5 (23.1)	0.82
Skin	< 5 (2.2)	< 5 (3.0)	–	NA
Urinary tract	–	–	–	NA
Viral	–	–	–	NA
Parents insurance status				0.56
Public	40 (86.9)	28 (84.8)	12 (92.3)	
Private	6 (13.1)	5 (15.2)	< 5 (7.7)	
Joint involvement				
Knee	18 (39.1)	14 (42.4)	<5 (30.8)	
Ankle/foot	10 (21.7)	8 (24.2)	<5 (15.4)	
Hip	<5 (6.5)	<5 (9.1)	–	
Wrist/hand	<5 (6.5)	<5 (6.1)	<5 (7.7)	
Shoulder	<5 (4.3)	<5 (3)	<5 (7.7)	
Polyarticular	10 (21.7)	5 (15.2)	5 (38.5)	
Methylprednisolone pulse pulse given	22 (47.8)	14 (42.4)	8 (61.5)	0.27
Biologic drug	6 (13.1)	6 (18.2)	–	NA
Joint aspiration performed	20 (43.5)	19 (57.5)	< 5 (7.7)	0.02
Length of hospital stay	4 (2–15)	4 (2–15)	3 (2–15)	0.56

Numbers reflect median with interquartile range or number with percentage

Characteristics of the S-JIA patients (Table 1) indicated that median age at onset (6.5 years; IQR 3.8–11) was similar for girls and boys and no patient identified as Indigenous, while 6% of female patients were born in Southeast Asia. Joint involvement was predominantly oligoarticular in lower extremity (67%) with less frequent polyarticular (22%) or upper extremity involvement (11%). However, polyarticular disease was more frequent in boys. During a median hospital stay of four days joint aspiration was performed more frequently in girls, while nearly half of all patients received intravenous (iv) pulse steroids (*n* =) and 18.2% (6/33) girls received biological drugs. Readmission within 31 days after discharge for the initial S-JIA episode occurred in four girls (12.2%) and five boys (38.5%) because of infections (18.1%) and disease flare (81.8%) requiring iv pulse steroid in seven (63.7%).

All patients were alive in WA at the end of the study period after a median follow up of 8 years (IQR 3–10) (Table 2). Over 307 person years of follow-up, 11 patients (23.9%) had no further need for hospital admission after their index SJIA admission and 35 patients (76.1%) required at least one further admission during follow-up. Among these 35 patients, 14 had at least one primary admission for sJIA for a total of 90 registered flares and an overall S-JIA flare rate of 29.3/100 person years with higher rates observed in boys than girls (58.3; CI 44.5–74.9 vs 14.7; CI 9.9–20.9) (*p* < 0.001) (Table 2). The remaining 21 patients had at least one non-sJIA admission with higher admission rates for non-sJIA complications in girls than boys (103.3 vs 54.4, *p* < 0.001), mainly due to a much higher frequency of diverse admissions reasons (Table 2). A further 41 joint aspirations were performed during follow-up while < 5 patients (4.3%) required hip arthroplasty after a mean period of 72 months. Notably, no diagnosis of diabetes mellitus or osteoporosis was recorded throughout the follow up period although three forearm fractures occurred. Overall, 33 patients (80.5%) had at least on ED visit post discharge with frequency and ED visit rates similar for girls and boys (Table 2).

**Table 2:** Patient characteristics during follow-up beyond one month after discharge for incident systemic juvenile idiopathic arthritis

	All (*n* = 46)	Girls (*n* = 33)	Boys (*n* = 13)	*p* value
Follow up (years)	8 (3–10)	7 (3–9.5)	9.5 (4–12)	0.32
Total person years	307	204	103	–
Nr patients ever readmitted	35 (76.1)	26 (78.8)	9 (69.2)	0.78
For S-JIA flare	14 (30.4)	8 (24.4)	6 (46.2)	0.24
Nr readmissions for S-JIA flare	90 (29.9)	30 (13.9)	60 (69.8)	< 0.001
S-JIA flare rate	29.3 (23.7–35.8)	14.7 (9.9–20.9)	58.3 (44.5–74.9)	< 0.001
Nr non S-JIA admissions	265 (46.8)	209 (49.3)	56 (39.4)	
For other MSK condition	58 (21.8)	45 (21.5)	15 (26.8)	
For digestive condition	18 (6.8)	7 ( 3.3)	11 (19.6)	
For respiratory condition	16 (6.0)	11 (5.3)	5 (8.9)	
For infectious condition	14 (5.3)	7 (3.3)	7 (12.5)	
For mental condition	7 (2.7)	5 (2.4)	<5 (3.6)	
For other condition*	152 (57.3)	134 (64.1)	16 (28.5)	
Non S-JIA admission rate	86.3 (76.4–97.2)	103.5 (89.1–117.3)	54.4 (41.1–70.6)	< 0.001
Nr patients with ED visits	33 (80.5)	21 (72.4)	11 (91.6)	0.24
Nr patients with > 1 ED visit	21 (51.2)	14 (48.3)	7 (63.6)	0.32
Total nr ED visits	225	138	87	
For abnormal findings	32 (14.2)	21 (15.2)	11 (12.6)	
For injury/poisoning	27 (12)	17 (12.3)	10 (11.5)	
For MSK condition	25 (11.1)	7 (5.1)	18 (20.7)	
For respiratory condition	21 (9.3)	13 (9.4)	8 (9.2)	
For infectious condition	17 (7.5)	11 (7.9)	6 (6.9)	
For digestive condition	12 (5.3)	8 (5.8)	<5 (4.6)	
ED visit rate	73.3 (64.2–83.4)	67.7 (56.8–79.9)	84.5 (67.7–100.4)	0.11

Figures indicate numbers (%), median with interquartile range or rates/100 person years with 95% confidence intervals. Small numbers given as <5 as required by HREC

*S-JIA* systemic juvenile idiopathic arthritis, *MSK* musculoskeletal conditions, *ED* emergency department

*Includes injury, ear/nose/throat conditions, allied health service, pregnancy, skin/eye and genitourinary disorders

## Discussion

We found a stable S-JIA incidence over 15 years in WA leading to a point prevalence of 7.15/100,000. We found no apparent association with preceding infections and S-JIA relapse requiring hospitalisation occurred in 14/46 patients (24%) with higher relapse rates in boys. Non S-JIA related admissions and ED visit rates were high throughout the follow up period period, including for patients with a monocyclic course.

Our data indicate that S-JIA is a rare condition in WA with an incidence that is stable over time and in line with European studies and the pooled incidence of 0.6/100.000 reported in a systematic review [10–13]. S-JIA in this region is thus three times more frequent than its adult counterpart ie adult-onset Still’s disease [3]. The 2014-point prevalence for S-JIA in this population study (7.15/100.000) was higher than the 2.7 to 6.4/100.000 prevalence in a systematic review [13], which may be explained by the inclusion of many pre-biologic studies in the systematic review as an improved prognosis has been observed over the last 2 decades [1, 14]. There are few data on seasonality for S-JIA and while the majority of S-JIA patients first presented during seasons associated with increased upper airway infection rates [15], we could not confirm a link with infections.

Median age at S-JIA diagnosis (6.5 years) falls with the reported range of 4.5 to 12 years in cohort studies [16, 17]. Our cohort contained a higher proportion of girls as in the original paper by Sir George Still [18] and the US cohort study [19] although other papers report gender equivalence [16, 17]. In hospital management included a high rate of intravenous corticosteroid therapy (48%) and use of a biological agent in almost a fifth of patients, in line with current and proposed therapy strategies to rapidly reduce inflammatory burden [20, 21]. The disease course was considered monocyclic in 69% of patients, although we cannot exclude that minor disease flares have occurred in these patients and treated on an outpatient basis. Patients that developed persistent/recurrent disease required frequent readmission in line with single centre studies [20, 22, 23]. Among these patients, rates for disease flares requiring admission were significantly higher in boys. The reasons for this are not clear and may include more severe disease in boys, possibly relate lower compliance with required maintenance therapy for persistent S-JIA [1, 24]. While we observed relatively few long-term complications, joint replacement was needed in 4% of patients confirming the continued potential for joint destruction with uncontrolled disease [2]. S-JIA patients often require corticosteroid therapy with subsequent tapering according to clinical findings [25, 26] and while we have no detailed medication data, the lack of development of confirmed diabetes be mellitus and osteoporosis suggest that high dose corticosteroid therapy was unlikely to longstanding in this cohort, possibly connected to the early and frequent use of biologicals. Infections accounted for 5% of all subsequent admissions and 7.5% of ED visits, which is largely in line with the rate of 5/100 person years observed in the German biologicals registry and 6.6% in a clinical trial setting [27, 28].

The limitations of this study should be recognised. While most S-JIA patients will be admitted to hospital to exclude serious infections and malignancy, we may have missed some S-JIA patients diagnosed/treated on an outpatient basis only. Thus, our incidence and prevalence data should be considered minimum estimates. Our S-JIA patients were identified based on the discharge diagnosis provided by the attending physicians, mainly paediatric rheumatologists. The lack of more granular clinical and laboratory findings did not allow formal testing against S-JIA classification criteria but the WARDER data set has 80–90% accuracy for various rheumatic diseases [3]. Similarly, the lack of clinical and biochemical disease activity measures and medication details precluded in depth analyses of disease severity such as MAS occurrence and treatment responses. The strength of this study is the use of data from a validated population-wide database with good diagnostic accuracy, reliable data linkage and long-term follow-up to determine health care outcomes in a rare disease.

## Conclusion

S-JIA incidence and prevalence in Western Australia aligns with European and US data. The disease was monocyclic in 42% with a flare rate of 29.3/100 person years in remaining patients. Arthroplasty (4%) was the main long term disease complication, but there was an overall continued high usage of hospital resources for non S-JIA conditions, including by patients with monocyclic disease.

## Supplementary Information

Below is the link to the electronic supplementary material.

Supplementary file1 (DOCX 32 KB)

## Data Availability

Approval for use of de-identified data was obtained from the Human Research Ethics Committee at the WA Department of Health (WADOH HREC# 2016.24). As this study was considered low risk by the WA Health HREC and due to the de-identified nature of the linked health data set, the requirement for patient consent was waived. WA Health is proprietor of this administrative health data dataset. Restrictions apply to the availability of these data, which were used under license of WA Health Data Linkage Branch for the current study. Data are however available from the authors upon reasonable request and after permission of WA Health and WA Data Linkage Branch.

## References

[1] Lee JJY, Schneider R (2018). Systemic juvenile idiopathic arthritis. Pediatr Clin North Am.

[2] Shenoi S, Wallace CA (2016). diagnosis and treatment of systemic juvenile idiopathic arthritis. J Pediatr.

[3] Nossent J (2022). Adult-onset Still’s disease in Western Australia: epidemiology, comorbidity and long-term outcome. Int J Rheum Dis.

[4] Hinze CH (2018). Practice and consensus-based strategies in diagnosing and managing systemic juvenile idiopathic arthritis in Germany. Pediatr Rheumatol Online J.

[5] Qu H (2021). Immunoprofiling of active and inactive systemic juvenile idiopathic arthritis reveals distinct biomarkers: a single-center study. Pediatr Rheumatol Online J.

[6] Mallalieu NL (2019). Intravenous dosing of tocilizumab in patients younger than two years of age with systemic juvenile idiopathic arthritis: results from an open-label phase 1 clinical trial. Pediatr Rheumatol Online J.

[7] Mejbri M (2020). Interleukin-1 blockade in systemic juvenile idiopathic arthritis. Paediatr Drugs.

[8] Klotsche J (2016). Outcome and trends in treatment of systemic juvenile idiopathic arthritis in the German national pediatric rheumatologic database, 2000–2013. Arthritis Rheumatol.

[9] Cimaz R (2016). Systemic-onset juvenile idiopathic arthritis. Autoimmun Rev.

[10] Moe N, Rygg M (1998). Epidemiology of juvenile chronic arthritis in northern Norway: a ten-year retrospective study. Clin Exp Rheumatol.

[11] Berntson L (2003). Incidence of juvenile idiopathic arthritis in the Nordic countries. A population based study with special reference to the validity of the ILAR and EULAR criteria. J Rheumatol.

[12] Cardoso I (2021). Age and sex specific trends in incidence of juvenile idiopathic arthritis in Danish birth cohorts from 1992 to 2002: a nationwide register linkage study. Int J Environ Res Public Health.

[13] Thierry S (2014). Prevalence and incidence of juvenile idiopathic arthritis: a systematic review. Jt Bone Spine.

[14] Ambler WG (2022). Refractory systemic onset juvenile idiopathic arthritis: current challenges and future perspectives. Ann Med.

[15] Fisman DN (2007). Seasonality of infectious diseases. Annu Rev Public Health.

[16] Hoeg PE (2022). Evaluation of macrophage activation syndrome in patients with systemic juvenile idiopathic arthritis: a single center experience. Int J Rheumatol.

[17] Marques ML (2022). Systemic autoinflammatory diseases in pediatric population. Asia Pac Allergy.

[18] Still GF (1897). On a form of chronic joint disease in children. Med Chir Trans.

[19] Janow G (2016). The systemic juvenile idiopathic arthritis cohort of the childhood arthritis and rheumatology research alliance registry: 2010–2013. J Rheumatol.

[20] Barut K (2019). Prognosis, complications and treatment response in systemic juvenile idiopathic arthritis patients: a single-center experience. Int J Rheum Dis.

[21] Nigrovic PA (2018). Bayesian comparative effectiveness study of four consensus treatment plans for initial management of systemic juvenile idiopathic arthritis: FiRst-Line Options for Systemic juvenile idiopathic arthritis Treatment (FROST). Clin Trials.

[22] Cakan M (2020). The frequency of macrophage activation syndrome and disease course in systemic juvenile idiopathic arthritis. Mod Rheumatol.

[23] Nigrovic PA (2014). Review: is there a window of opportunity for treatment of systemic juvenile idiopathic arthritis?. Arthritis Rheumatol.

[24] Correll CK, Binstadt BA (2014). Advances in the pathogenesis and treatment of systemic juvenile idiopathic arthritis. Pediatr Res.

[25] Kearsley-Fleet L (2019). Short-term outcomes in patients with systemic juvenile idiopathic arthritis treated with either tocilizumab or anakinra. Rheumatology (Oxford).

[26] Onel KB (2022). 2021 American college of rheumatology guideline for the treatment of juvenile idiopathic arthritis: therapeutic approaches for oligoarthritis, temporomandibular joint arthritis, and systemic juvenile idiopathic arthritis. Arthritis Rheumatol.

[27] Klein A (2020). Long-term surveillance of biologic therapies in systemic-onset juvenile idiopathic arthritis: data from the German BIKER registry. Rheumatology (Oxford).

[28] Kimura Y (2017). Pilot study comparing the childhood arthritis & rheumatology research alliance (CARRA) systemic juvenile idiopathic arthritis consensus treatment plans. Pediatr Rheumatol Online J.

