# Supplementary material for: Systemic juvenile idiopathic arthritis: frequency and long-term outcome in Western Australia
Source: Rheumatol Int. 2023 Mar 29;43(7):1357–62. doi: 10.1007/s00296-023-05318-1 (PMC10185593; doi:10.1007/s00296-023-05318-1)

| Condition | Coding |
| --- | --- |
|  |  |
| Systemic juvenile arthritis (S-JIA) | M08.20-M08.29 |
| Diabetes Mellitus | E08.0-E013.9 |
| Serious infections | J. Rheumatology, 2020; 47:424-30 |
| Osteoporosis | M81.0 - M81.8 |
| Fractures | S02,S12,S22,S32,S42,S52,S62,S72,S82 S92 |
| ACHI codes for procedures: |  |
| Arthrocentesis | 50124 |
| Methyl pred iv pulse | 92188.00, 96197-03,96199-03 |
| Biologic infusion | 96199-00, 96199-09 |
| Joint replacement | 46300-01 through 50127-00 |
|  |  |

Suppl Table 1 Codes used for classifying patients and complications using ICD10-AM and Australian Classification of Health Interventions terminology.

Suppl Figure 1 Seasonality of S-JIA diagnosis


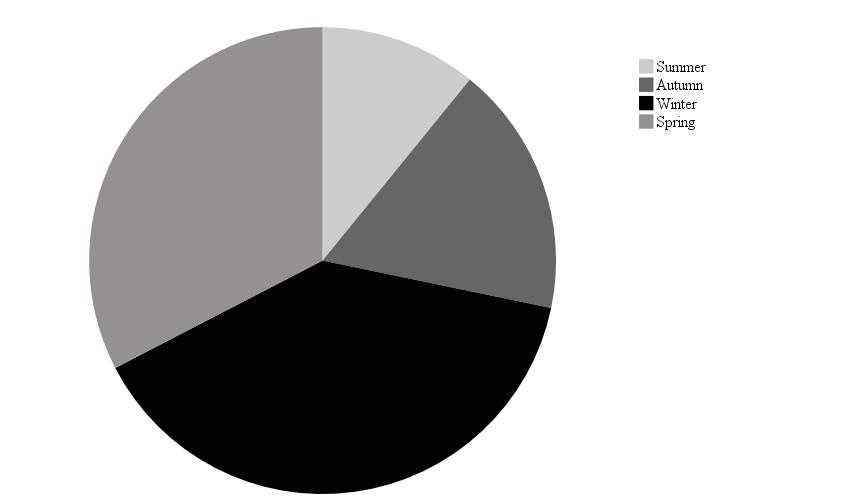

Supplement: Supplementary file 1 — Supplementary file1 (DOCX 32 KB) [file 296_2023_5318_MOESM1_ESM.docx]
